# Oxygen Saturation Targeting During Delivery Room Stabilization: What Does This Mean for Regional Cerebral Oxygenation?

**DOI:** 10.3389/fped.2019.00274

**Published:** 2019-07-02

**Authors:** Corinna Binder-Heschl, Gerhard Pichler, Alexander Avian, Bernhard Schwaberger, Nariae Baik-Schneditz, Lukas Mileder, Stefan Heschl, Berndt Urlesberger

**Affiliations:** ^1^Department of Pediatrics, Division of Neonatology, Medical University of Graz, Graz, Austria; ^2^Research Unit for Neonatal Micro- and Macrocirculation, Department of Pediatrics, Medical University of Graz, Graz, Austria; ^3^Institute for Medical Informatics, Statistics and Documentation, Medical University of Graz, Graz, Austria; ^4^Department of Anesthesiology and Intensive Care Medicine, Pediatric Anesthesia, Medical University of Graz, Graz, Austria

**Keywords:** near infrared spectroscopy, regional cerebral oxygenation, respiratory support, transition period, preterm neonates, arterial oxygen saturation, target saturation

## Abstract

**Objective:** To investigate if preterm neonates with arterial oxygen saturation (SpO_2_)<80% at 5 min after birth show different regional cerebral tissue oxygen saturation (rcStO_2_), compared to infants reaching the target.

**Methods:** Retrospective analysis of four prospective observational studies. Preterm neonates needing respiratory support during delivery room stabilization were included. Regional cerebral tissue oxygen saturation was measured with near-infrared spectroscopy (NIRS) during the first 15 min after birth along with SpO_2_ and heart rate (HR). Neonates were divided into two groups: those with a 5-min SpO_2_ ≥ 80% (“≥80% group”) and those with a 5-min SpO_2_ < 80% (“<80% group”). Groups were compared regarding rcStO_2_, SpO_2_, and HR. Furthermore, we analyzed whether a 5-min SpO_2_ < 80% was associated with a rcStO_2_ below the 10th percentile at the same time point.

**Results:** 146 neonates were included, with 68 (47%) in the “≥80% group” and 78 (53%) in the “<80% group.” Neonates in the “ <80% group” had a significantly lower rcStO_2_ (*p* < 0.001). Furthermore, 80.3% of neonates in the “ <80% group” and 23.4% in the “≥80% group” had rcStO_2_ values below the 10th percentile at 5 min (*p* < 0.001). HR was significantly lower at minute 3 and 4 in the “ <80% group” (*p* < 0.002).

**Conclusion:** Preterm infants needing respiratory support, who do not reach the SpO_2_ target of 80% at 5 min after birth, show significantly diminished rcStO_2_ values compared to neonates reaching the target.

## Introduction

Within the last years a significant amount of research has been undertaken trying to understand the physiological processes during neonatal transition. The course of arterial oxygen saturation (SpO_2_) during the first minutes after birth became a topic of high interest in neonatal medicine, as this seems to be crucial for the infant's further outcome. Pulse oximetry monitoring in the delivery room is now recommended in order to gain more information about the neonate's condition and to guide respiratory and supplemental oxygen support (1, 2). Percentiles of postnatal rise in SpO_2_ were obtained from studies including healthy term and preterm infants without medical intervention (3).

In infants with need for respiratory support, adequate SpO_2_ targeting is very much under debate. The European Resuscitation Council and the American Heart Association published recommendations for threshold values for certain time points within the postnatal stabilization period (1, 2, 4, 5). Besides SpO_2_ targets, the ideal strategy of initial oxygen supplementation for delivery room resuscitation is not clear yet, and recommendations have changed substantially within the last decade (6). There seems to be a narrow range for SpO_2_ targeting, as preterm infants are at risk of organ injury due to biochemical oxidative stress on the one hand, but also at risk for hypoxic injury due to respiratory insufficiency on the other hand (7–11). Recently, a study by Oei et al. showed that preterm infants initially resuscitated with room air (21% oxygen) had an increased risk of hospital death compared to infants resuscitated with 100% oxygen. Additionally, they showed that in preterm neonates, not reaching a 5-min SpO_2_ of 80%, risk of mortality increased (12, 13). The same authors performed a meta-analysis of eight randomized controlled oxygen titration trials and demonstrated that almost half of the preterm infants did not reach a 5-min SpO_2_ of 80%. Moreover this was associated with a higher risk of developing bradycardia and intraventricular hemorrhage (IVH) (13). The brain of preterm infants is particularly vulnerable to hypoxia. However, monitoring SpO_2_ and heart rate (HR) by pulse oximetry may not provide adequate information about the oxygen supply to the brain, as oxygen delivery depends on SpO_2_ and organ perfusion (14).

A common method to measure regional cerebral oxygenation is near-infrared spectroscopy (NIRS). This non-invasive method can be used in preterm infants during delivery room stabilization (14). Moreover, reference ranges and centile charts of regional cerebral oxygenation during the first 15 min after birth are available for term and preterm infants without medical support (15, 16). A few years ago, a study showed that preterm neonates developing IVH within the first days after birth had a significantly lower regional cerebral oxygenation during immediate transition (14, 17). This association was also highlighted by Katheria et al. recently (18). Moreover, a randomized Phase I/II pilot trial demonstrated that monitoring regional cerebral oxygenation during neonatal resuscitation in combination with dedicated interventions reduced the burden of cerebral hypoxia and thus may be useful in guiding respiratory and supplemental oxygen support after birth (19).

Hence, the aim of the present study was to investigate whether reaching a SpO_2_ target of 80% at 5 min after birth was associated with a significantly different course of regional cerebral oxygenation.

## Methods

This study represents a retrospective analysis of four prospective observational studies, conducted between December 2010 and March 2017 at the Division of Neonatology, Department of Pediatrics and Adolescent Medicine, Medical University of Graz, Austria (19–22).

We included preterm infants, <37 weeks of gestation, who fulfilled all of the following criteria: (1) decision to conduct full life support, (2) obtained written, informed consent from the parents prior to birth, (3) need for respiratory support during immediate transition in the delivery room, and (4) no congenital malformations, which could affect the oxygenation or mortality. Only neonates after cesarean section were included to avoid a delay or disturbance of immediate mother-baby skin-to-skin contact in vaginally born neonates. According to the local clinical routine cord clamping was performed up to 30 s after birth, and neonates were stabilized according to the current resuscitation guidelines (2, 5, 6, 23, 24).

The Regional Committee on Biomedical Research Ethics approved all of the included studies.

In all infants maternal medical history and neonatal demographic data, as gestational age, birth weight, Apgar scores and pH of the umbilical artery, were documented.

The included studies were designed to measure regional cerebral oxygenation during the first 15 min after birth, using NIRS. A standardized protocol was followed in all studies: the study period started when the cord was clamped and lasted for 15 min. After the cord was clamped, the midwife took the neonate to the resuscitation table (CosyCot™; Fisher& Paykel Healthcare; New Zealand), placing the infant in supine position under a pre-warmed overhead heater. If the neonate was <28 weeks of gestation, its body was placed into a polyethylene bag. During clinical observation/ stabilization by a neonatologist a member of the research group attached a NIRS sensor to the infant's left forehead, using a gauze bandage without disturbing routine medical care. Furthermore, a pulse oximetry sensor (IntelliVue MP50 monitor; Philips; Netherland) was applied pre-ductal, on the right palm or wrist, to monitor SpO_2_ and HR. All variables were automatically recorded and stored continuously in a multichannel system “alpha-trace digital MM” (BEST Medical Systems; Austria) for subsequent analysis. SpO_2_ and HR values were stored every second.

If obstruction of the upper airway was obvious, immediate suction of the oropharynx was performed. Respiratory support, as continuous positive airway pressure (CPAP) and/or positive pressure ventilation (PPV), was provided via an appropriate round silicone face mask (LSR Silicon mask no. 0/0 or 0/1; Laerdal; Norway) and the “Neopuff Infant T- Piece Resuscitator” (Perivent; Fisher& Paykel Healthcare; New Zealand). The default settings were a gas flow of 6–8 L/min, positive end expiratory pressure of 5 cmH_2_O, peak inspiratory pressure of 25–30 cmH_2_O and a fraction of inspired oxygen (FiO_2_) of 0.3. To measure FiO_2_ the Florian Neonatal Respiratory Function Monitor (Acutronic Medical Systems AG; Switzerland) was used.

Depending on the study, either the INVOS 5100C (INVOS cerebral/ somatic oximeter monitor; Medtronic; Minneapolis, USA) with a neonatal sensor or the NIRO 200-NX tissue oxygenation monitor (Hamamatsu Photonics; Hamamatsu City, Japan) was used to measure the cerebral regional oxygen saturation (crSO_2_) or cerebral tissue oxygenation index (cTOI), respectively. The sample rate of crSO_2_ was 0.13 Hz and of cTOI 2 Hz. Within this manuscript crSO_2_ and cTOI measurements have been combined for statistical analysis and are named regional cerebral tissue oxygen saturation (rcStO_2_).

Cerebral fractional tissue oxygen extraction (cFTOE) was calculated for each minute [(SpO_2_-rcStO_2_)/SpO_2_].

According to the SpO_2_, infants were divided into two groups: those with a 5-min SpO_2_ ≥ 80% (“≥80% group”) and those with a 5-min SpO_2_ <80% (“ <80% group”). Both groups were compared to each other regarding demographic data, rcStO_2_, SpO_2_, cFTOE, FiO_2_, and HR.

Furthermore, we analyzed whether not reaching an SpO_2_ of 80% at 5 min after birth was associated with a rcStO_2_ below the 10th percentile at the same time point (15, 16).

### Statistical Analysis

Baseline characteristics are presented as mean ± standard deviation for normally distributed variables or median (range) when the distribution was skewed. The presented values of rcStO_2_, SpO_2_, HR, cFTOE, and FiO_2_ are means for a whole minute. Categorical variables are given with numbers and percentage. To compare demographic data between groups, a Mann-Whitney-U-test was used for non-parametric data, a Student's *t*-test for parametric data or a *X*^2^-test for categorical measurements. In the main analysis we investigated the changes in rcStO_2_, SpO_2_, HR, and cFTOE within the first 15 min after birth using a linear mixed model with a fixed effect for time and group (“ <80% group” vs. “≥80% group”) and a first-order autoregressive covariance structure. Since rcStO_2_ and cFTOE were measured using two different devices, a further fixed effect (device: INVOS 5100C vs. NIRO 200-NX) was included for this analysis. If for an infants measurements both devices were available, INVOS 5100C values were used for statistical analysis. Results according to these linear mixed models are presented using means and 95% confidence intervals (95% CI). A *p*-value < 0.05 was considered statistically significant. The statistical analysis were performed using IBM SPSS Statistics 24 (IBM Corporation; Armonk; New York; USA).

## Results

**Figure 1:**
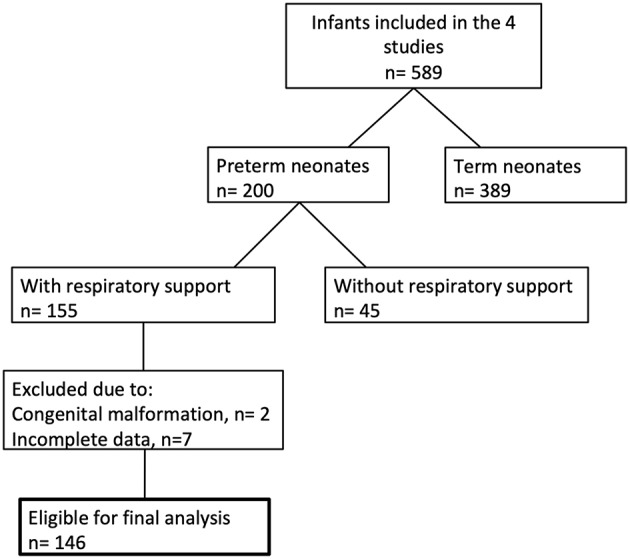
Flow diagram showing the number of included infants.

Between December 2010 and March 2017, 146 preterm neonates fulfilled the entry criteria (Figure 1). In 68 infants (47%) the SpO_2_ was ≥80% 5 min after birth, whereas 78 infants (53%) had SpO_2_ values <80% at the same time point. Demographic data of the groups are shown in Table 1. The most immature infant in the “≥80% group” was 23 + 3 and in the “ <80% group” 23 + 6 weeks of gestational age. Our analysis includes 52 infants below a gestational age of 32 weeks.

**Table 1:** Patient demographic characteristics.

	**“≥80% group”** ***n* = 68**	**“ <80% group”** ***n* = 78**	**Group comparison** ***p*-value**
Gestational age, weeks	32.0 (23.4–36.2)	33.0 (23.8–36.6)	**0.009**
Birth weight, g	1,484 (466–3,130)	1,880 (550–3,105)	**0.001**
Apgar 1	8 (3–9)	8 (3–9)	0.363
Apgar 5	9 (6–10)	8 (6–10)	**0.001**
Apgar 10	9 (8–10)	9 (7–10)	0.315
pH umbilical artery^#^	7.31 ± 0.05	7.31 ± 0.04	0.601
Male sex^*^	34 (50)	32 (41)	0.277

Data are presented as median (range), unless indicated

# *mean ± SD*,

* *n (%)*.

*Bold numbers are statistically significant (p < 0.05)*.

None of the neonates died during the study period. In the “≥80% group” 6 (4.1%) and in the “ <80% group” 7 (5.5%) infants had to be intubated during the study period (no significant difference, *p* = 0.97). Three infants died before hospital discharge, 1 (0.7%) in the “≥80% group” and 2 (1.6%) in the “ <80% group” (no significant difference, *p* = 0.64). The infant in the “≥80% group” died after 5 months in the neonatal intensive care unit due to severe bronchopulmonary dysplasia. The two neonates in the “ <80% group” died because of redirection of care. In one case it was due to the parents' specific request and in the other case due to bilateral severe intraventricular hemorrhage. No other infant had severe intraventricular hemorrhage.

### Arterial Oxygen Saturation (SpO_2_) and Heart Rate (HR)

**Figure 2:**
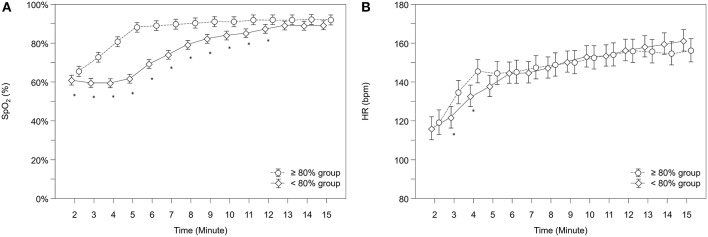
Courses of **(A)** arterial oxygen saturation (SpO_2_) and **(B)** heart rate (HR) within both groups. Significant differences between groups (*p* < 0.05) for each minute are marked with *; Data are means and 95% CI. Data points are slightly offset on the x-axis for visual reasons.

Over the study period, SpO_2_ was significantly lower in the “ <80% group” compared to the “≥80% group” (*p* < 0.001) and showed significantly different courses over time (*p* < 0.001). After *post-hoc* analysis for group differences at each minute, the “ <80% group” had significantly lower SpO_2_ values from minute 2 to 12 (Figure 2A). At 5 minutes after birth, the mean SpO_2_ in the “ <80% group” was 62% (95%CI: 60–64%) versus 88% (95%CI: 86–91%) in the “≥80% group.”

Neonates showed significantly different courses over time in their HR (*p* = 0.041). *Post-hoc* analysis for group differences at each minute showed that the “ <80% group” had significantly lower HR values from minute 3 to 4 (Figure 2B). In total, 8 infants (5.8%) had HR values below 100 bpm at 5 min of age, 6 (8.2%) in the “ <80% group” and 2 (3.0%) in the “≥80% group,” but group differences didn't reach statistical significance (*p* = 0.280).

### Regional Cerebral Tissue Oxygen Saturation (rcStO_2_)

**Figure 3:**
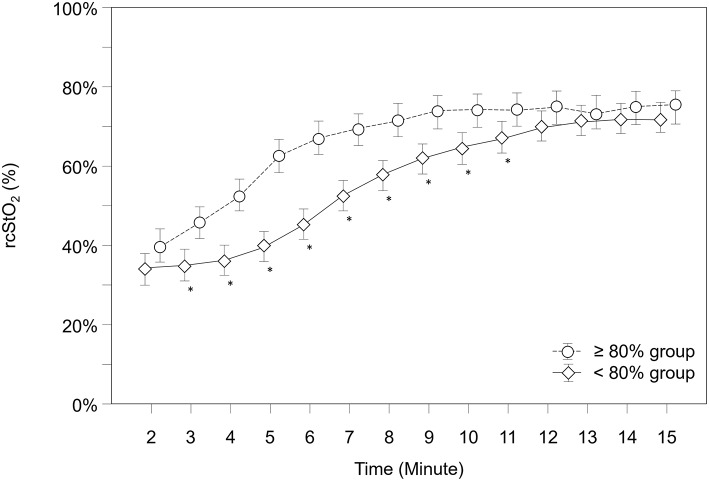
Courses of regional cerebral tissue oxygen saturation (rcStO_2_) within both groups. Significant differences between groups (*p* < 0.05) for each minute are marked with *; Data are means and 95% CI. Data points are slightly offset on the x-axis for visual reasons.

Over the whole study period, rcStO_2_ was significantly lower in the “ <80% group” compared to the “≥80% group” (*p* < 0.001) and showed significantly different courses in both groups over time (*p* < 0.001). *Post-hoc* analysis for group differences at each minute showed that the “ <80% group” had significantly lower rcStO_2_ values from minute 3 to 11 (Figure 3).

Further analysis showed that 49 neonates (76.6%) in the “≥80% group,” but only 14 neonates (19.7%) in the “ <80% group” had rcStO_2_ values above the 10th percentile at 5 min after birth (*p* < 0.001) (15, 16). A subgroup analysis for each device (INVOS 5100C and NIRO 200-NX) did show same results, therefore data are not presented separately.

### Cerebral Fractional Tissue Oxygen Extraction (cFTOE)

**Figure 4:**
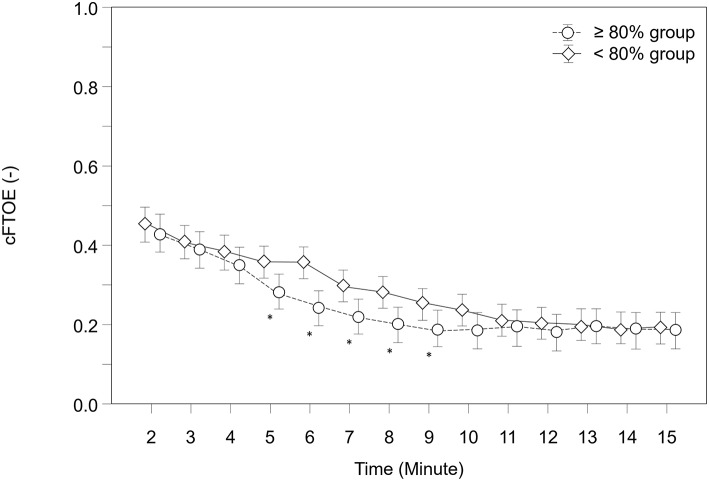
Courses of cerebral fractional tissue oxygen extraction (cFTOE) within both groups. Significant differences between groups (*p* < 0.05) for each minute are marked with *; Data are means and 95% CI. Data points are slightly offset on the x-axis for visual reasons.

Over the study period, cFTOE showed a tendency to be lower in the “≥80% group” (*p* = 0.065). Neonates in both groups showed significant different courses over time (*p* = 0.007). A *post-hoc* analysis for group differences at each minute revealed that infants in the “≥80% group” had significantly lower cFTOE values from minute 5 to 9 (Figure 4).

### Fraction of Inspired Oxygen (FiO_2_)

**Figure 5:**
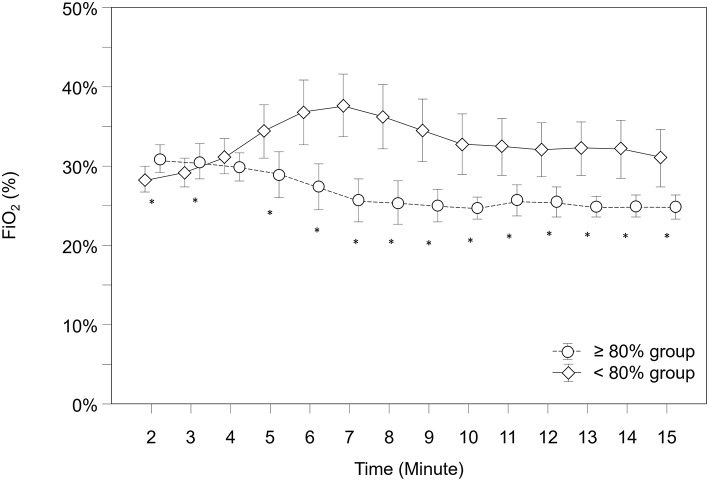
Courses of fraction of inspired oxygen (FiO_2_) within both groups. Significant differences between groups (*p* < 0.05) for each minute are marked with *; Data are means and 95% CI. Data points are slightly offset on the x-axis for visual reasons.

Over the study period, FiO_2_ was significantly higher in the “ <80% group” compared to the “≥80% group” (*p* < 0.001) and showed significantly different courses over time (*p* < 0.001). According to *post-hoc* analysis for group differences at each minute, the “ <80% group” had significantly lower FiO_2_ values from minute 2 to 3 and higher values from minute 5 to 12 (Figure 5).

## Discussion

The present study is a retrospective analysis, which, by combining detailed course of SpO_2_ and rcStO_2_, presents a comprehensive overview on oxygenation status of preterm infants needing respiratory support immediately after birth. In contrast to Oei et al. (12) (initial FiO_2_ 0.21 vs. 1.0) we routinely started with an FiO_2_ of 0.3 during postnatal stabilization, nevertheless only 47% of the preterm infants reached the SpO_2_ target of 80% at 5 min of age. Moreover, we observed that preterm neonates not reaching a SpO_2_ of 80% 5 min after birth had significantly lower rcStO_2_ values over the whole study period.

In our study the behavior of SpO_2_ was similar to a previous meta-analysis of eight randomized controlled oxygen titration trials, which showed that almost half of the enrolled preterm infants did not reach the target SpO_2_ of 80% 5 min after birth. The authors already raised the question whether recommended SpO_2_ targets are reached with the current oxygen supplementation strategies (13). The meta-analysis focused on 5-min SpO_2_ values, whereas the present study observed the course of SpO_2_ over the first 15 min after birth. The differences between the two groups in our study already occurred very early, in fact from minute 2 onwards. It also took more than 10 min until both groups had similar values, though the mean SpO_2_ of the “ <80% group” did not reach 90% at any stage of the study period. Thus, using an initial FiO_2_ of 0.3, the results of the present analysis are similar to those from a recent RCT (comparing room air vs. FiO_2_ of 1.0 for initial delivery room resuscitation) (12). Furthermore, Rabi et al. compared the outcome of extremely preterm infants before and after the Canadian resuscitation guidelines were changed (from using FiO_2_ of 1.0 to using lower oxygen concentration) and found a higher risk of death and severe neurological injury in the group with low supplemental oxygen (25).

In regard to FiO_2_, both groups started with a default setting of 0.3. Nevertheless, we would like to point out, that our FiO_2_ data are measured data (using a respiratory function monitor), so they may vary slightly from set FiO_2_. During the first 4 min the FiO_2_ values were close to each other, starting with minute 5 the “ <80% group” showed significantly higher FiO_2_ values. Statistically the “ <80% group” had a significantly lower FiO_2_ during minute 2 and 3, but from a clinical point of view, both groups were still very close to each other.

It is known that SpO_2_ and regional cerebral oxygenation behave differently within the first minutes after birth. In fact regional cerebral oxygenation reaches a plateau earlier compared to SpO_2_, which may indicate a preferential oxygen delivery to the brain (26, 27). Oxygen delivery to the brain is affected not only by SpO_2_, but hemoglobin concentration and cerebral blood flow, which itself depends on cardiac output and vascular resistance. Thus, a priori it was not clear whether regional cerebral oxygenation was diminished in preterm infants presenting with a SpO2 <80%. Nevertheless, rcStO_2_ values of the present study were significantly reduced in the <80% group, emphasizing the point that oxygenation status of these preterm infants may be critically low or even too low. This is underlined by the fact that the <80% group had significantly diminished HR values too. In the present study it was not the gestational age (which was lower in “≥80%group”), but the clinical situation that influenced the oxygenation, as the “ <80% group” infants presented with significantly lower 5-min Apgar scores.

Dawson et al. defined percentiles for the rise of SpO_2_ within the first 10 min after birth in infants without any need for respiratory support (3). Nevertheless, in infants with need for respiratory support there is no evidence based data available for the selection of the best SpO_2_ target. In 2015 recommendations were published to target for a region equivalent the 25th percentile (4, 5).

To make NIRS more suitable for clinical routine, reference ranges, similar to the SpO_2_ percentile charts of Dawson et al. (3), have been established for term and preterm infants without medical intervention too (15, 16). Similar to SpO_2_ it still remains unclear, which regional cerebral oxygenation threshold should be targeted. In a previous study, our research group reported an associative relationship between the appearance of IVH during the first week after birth and low regional cerebral oxygenation values during immediate transition in preterm infants below 32 weeks of gestational age. Infants in the “IVH group” had significantly lower regional cerebral oxygenation values from minute 7 to 15 after birth compared to the “non-IVH group,” and moreover showed rcStO_2_ values below the 10th percentile (17). Therefore, rcStO_2_ values below the 10th percentile may be considered as worrisome. In the present study, 80.3% of infants in the “ <80% group” did show a rcStO_2_ below the 10th percentile, pointing out that these neonates did not only suffer from arterial hypoxia, but also from impaired regional cerebral oxygenation, which may contribute to perinatal brain injury. Moreover, we are concerned that even 23.4% of all infants in the “≥80% group” still showed rcStO_2_ values below the 10th percentile. The question remains whether we can influence regional cerebral oxygenation with our clinical actions. Recently, a multicenter randomized pilot-trial (COSCOD Trial) demonstrated that an additional monitoring of regional cerebral oxygenation in combination with clinical treatment guidelines was feasible, and that such an approach did reduce the burden of cerebral hypoxia during neonatal resuscitation (19). Furthermore, the trial proved that it was feasible to keep regional cerebral oxygenation values above the 10th percentile with the use of a dedicated clinical protocol. With the use of cerebral NIRS similar results have been described for preterm infants during the first 72 h by another randomized multicenter trial, the SafeboosC- trial. The study showed a reduction in the burden of cerebral hypoxia in extremely preterm infants when regional cerebral oxygenation was monitored and predefined treatment guidelines were used. Furthermore, the study found that low regional cerebral oxygenation during the first 3 days of life was associated with severe intracranial hemorrhage (28, 29). Thus, the present study does not only add further information to the topic of optimizing oxygenation during neonatal transition, but contributes to the possibility of using NIRS as a monitoring tool in that situation.

The present study has several limitations. Cerebral blood flow is regulated by cerebral vascular resistance, which is influenced by changes in paO_2_, paCO_2_, and blood glucose (30–32). There is evidence that cerebral blood flow correlates positively with paCO_2_ (33) and is increased by the presence of hypoglycemia (31). It even appears that paCO_2_ has more influence on cerebral blood flow than arterial blood pressure (34). In the present study no information on blood gas changes (except the arterial umbilical pH) or blood glucose levels was available, therefore we cannot interpret the potential influence of these parameters. Another influencing factor on oxygen delivery is hemoglobin, which was also not measured in our study. However, we presume that clinically relevant changes in hemoglobin concentration were unlikely, since the study period was only 15 min short, and no serious bleedings were observed. Secondly, the cord clamping time was similar in all infants.

As described above, we only included infants delivered by cesarean section. Therefore, we have no information, whether vaginal birth would have shown different results. Though it is known, that SpO_2_ and HR are lower in cesarean-delivered infants, no differences in cerebral oxygenation could be observed with respect to mode of delivery (27).

In the present study rcStO_2_ was measured with two different devices, the INVOS 5100C and the NIRO 200-NX. Since these two different devices provide systematically different values we included the type of device in the analysis to avoid an influence on the results. As expected this factor resulted in significant differences between these two devices (rcStO2: *p* = 0.002; cFTOE: *p* < 0.001). Although these factors were included in the analysis, it cannot be ruled out that the type of device may have an influence, which we have not modeled in our analysis. It is known that absolute values of different devices are not comparable one-to-one, but the differences in device calibration are well-defined and a study showed no significant differences in the mean regional tissue oxygenation between the two NIRS devices (35, 36). It needs to be considered that reference ranges for the NIRO 200-NX device were derived from term neonates without medical intervention, who might have a higher cTOI during transition period. The reference values for the INVOS 5100C device were derived from preterm neonates too.

In conclusion, in this retrospective observational analysis we observed that preterm neonates not reaching a SpO_2_ of 80% 5 min after birth had a significantly lower rcStO_2_ over the whole study period. Furthermore, 80.3% of infants in the “ <80% group” did show rcStO_2_ values below the 10th percentile, and we must point out that even 23.4 % of the neonates in the “≥80% group” still had rcStO_2_ values below the 10th percentile 5 min after birth. The ideal oxygen targeting and supplementation policy remains a matter of debate and needs further work. The results of the COSCOD Trial encourage the use of NIRS during neonatal resuscitation.

## Ethics Statement

All data included in this retrospective study was extracted from four different prospective studies. The ethics committee of the Medical University of Graz approved all four studies. Written informed consent was obtained from all parents in accordance with the Declaration of Helsinki.

## Author Contributions

CB-H and BU designed the study. CB-H, GP, NB-S, BS, and LM collected the data. AA and CB-H performed statistical analysis. CB-H, SH, and BU wrote the first draft. All the authors critically reviewed the manuscript and approved the final version submitted for publication.

### Conflict of Interest Statement

The authors declare that the research was conducted in the absence of any commercial or financial relationships that could be construed as a potential conflict of interest.

## Abbreviations

SpO_2_: Arterial oxygen saturation
cTOI: Cerebral tissue oxygenation index
crSO_2_: Cerebral regional oxygen saturation
HR: Heart rate
rcStO_2_: Regional cerebral tissue oxygen saturation
cFTOE: Cerebral fractional tissue oxygen extraction
NIRS: Near-infrared spectroscopy
IVH: Intraventricular hemorrhage
FiO2: Fraction of inspired oxygen.
